# Evaluating statistical methods to predict indoor black carbon in an urban birth cohort

**DOI:** 10.1016/j.indenv.2025.100084

**Published:** 2025-04-01

**Authors:** Sherry WeMott, Grace Kuiper, Sheena E. Martenies, Matthew D. Koslovsky, William B. Allshouse, John L. Adgate, Anne P. Starling, Dana Dabelea, Sheryl Magzamen

**Affiliations:** a Department of Environmental and Radiological Health Sciences, Colorado State University, Fort Collins, CO, USA; b Department of Statistics, Colorado State University, Fort Collins, CO, USA; c Department of Environmental and Occupational Health, Colorado School of Public Health, University of Colorado Anschutz Campus, Aurora, CO, USA; d Department of Epidemiology, Colorado School of Public Health, University of Colorado Anschutz Medical Campus, Aurora, CO, USA; e Lifecourse Epidemiology of Adiposity and Diabetes (LEAD Center), University of Colorado Anschutz Medical Campus, Aurora, CO, USA; f Department of Pediatrics, School of Medicine, University of Colorado Anschutz Medical Campus, Aurora, CO, USA; g University of Illinois Urbana-Champaign, Department of Health and Kinesiology, Urbana, IL, USA

**Keywords:** Indoor air pollution, Black carbon, Predictive modeling

## Abstract

Most air pollution epidemiology studies rely on outdoor exposure data from various sources, such as reference monitors, low-cost monitors, models, or Earth observations. However, people spend 90 % of their time indoors, with 70 % of that time spent at home, which may result in misclassification of air pollution exposure when using data reflecting ambient concentrations. In this study, we evaluated methods to predict residential indoor black carbon (BC) from outdoor BC, PM2.5, and housing characteristics to support future efforts in estimating personal air pollution exposure. Households from the Healthy Start cohort in Denver, CO hosted paired indoor/outdoor low-cost air samplers for one-week periods during spring 2018, summer 2018, and winter 2019. Participants completed questionnaires about housing characteristics like building type, flooring, and heating and cooling methods. Filters were analyzed for BC using transmissometry. Ridge, LASSO and ordinary least squares regression (OLS) techniques were used to build predictive models of indoor BC given the available set of covariates. Leave-one-out cross-validation was used to assess the predictive accuracy of each model. We hypothesized that Ridge and LASSO will obtain improved predictive performance over the OLS model due to regularization. A total of 27 households participated, with 39 paired measurements available after data cleaning. All winter data were excluded due to high variability and incomplete sampling times for outdoor measurements. Performance issues suggested insufficient weatherproofing of monitors for low temperatures. The Ridge regression showed the best predictive performance. The final inference model included outdoor PM2_.5_, hard floors, and the presence of pets in the home, accounting for approximately 28 % of the variability in indoor BC concentrations measured in participant homes. In the absence of indoor monitoring, household characteristics like flooring and the presence of pets can help predict indoor levels of BC.

## 1. Introduction

Although monitoring and regulation of air quality has improved in the last several decades, exposure to air pollution, from both ambient and indoor sources, is still a major public health problem worldwide [1]. The majority of air pollution epidemiology studies are predicated on outdoor exposure data, whether those data are from reference monitors, low-cost monitors, models, or Earth observations. However, people spend approximately 90 % of their time indoors, and of that, 70 % of the time is spent in homes [2]. Further, infants and young children spend a majority of their time indoors and at home [3]. Prior research indicates that personal levels of exposure, for PM2.5 especially, do not agree with area area-based measures from reference monitors [4] leading to possibilities of exposure misclassification when home environments are not accounted for [5]. Additional risk also exists for those in areas of lower socioeconomic status, as environmental hazards such as air pollution are often disproportionately distributed across populations [6]. Improved methods for indoor and ambient air pollution exposure assessment are critical to understand inequitable health burden and to protect vulnerable populations. Fine particulate matter (PM2.5) and its chemical constituents are commonly measured to estimate exposure to ambient air pollution. The constituents of PM2.5, which are source dependent, determine its chemical composition and influence its impact on health. For example, recent research has shown that PM2.5 from combustion sources may be more harmful than particulate matter from other sources [7]. One constituent of PM2.5, black carbon (BC), is the light-absorbing refractory form of elemental carbon. Elemental carbon (EC) consists of pure carbon in various forms; BC is the carbon remaining after pyrolysis or incomplete combustion and can be used as an indicator of exposure to traffic-related air pollution (TRAP). BC is also known to have a more heterogeneous spatial distribution than PM2.5, and therefore may be a more informative measurement at the intraurban scale [8]. In addition to the heterogeneous spatial distribution of BC, concentrations are also known to fluctuate seasonally, with higher concentrations often seen in winter. Air quality in winter can be impacted by an increase in biomass burning as well as meteorological conditions like colder, dryer air that traps more pollution [9].

There are several methods to estimate medium and long-term TRAP exposure, including techniques such as Land Use Regression (LUR) and dispersion modeling [10]. In these situations, regional ambient air pollution data is often extrapolated to estimate individual exposure; however, multiple studies have shown poor correlation between ambient PM2.5 measurements and personal exposure data [11,12]. Use of outdoor BC concentrations as a proxy for personal exposure also has limitations. The indoor environment plays a significant role in personal exposure to air pollution that is not accounted for using LUR and similar approaches to assess ambient air pollution exposure. Studies have shown that outdoor BC concentrations do not fully explain the variability in indoor BC levels [13,14]. Personal monitoring has been shown to be a better estimate of TRAP exposure [15] because air pollution exposure is measured in each microenvironment such as school, work, and transportation.This discrepancy highlights the need for more accurate methods to assess personal exposure to BC.

Previous studies demonstrate associations between BC and cancer, respiratory diseases, and cardiovascular dysfunction [16]. Accurate assessment of personal exposure to BC is crucial for understanding its health impacts. Particularly vulnerable populations such as the very young, elderly and those with cardiovascular and respiratory diseases may spend even more time inside. Unfortunately, representative samples are difficult to achieve for indoor air quality monitoring in residential settings, which requires compliance from multiple households as well as reliable instruments to measure indoor air pollution. In lieu of personal of indoor monitoring, statistical methods to predict indoor air quality may be useful for estimating individual air pollution exposure more accurately than using ambient air quality data alone.

Indoor air quality is influenced by the infiltration of outdoor PM_2.5_, including BC, as well as sources of indoor air pollution, such as cooking on unvented stoves, burning candles, smoking cigarettes and burning solid fuels for heating. Infiltration and exfiltration occur when air moves between indoor and outdoor environments through unintentional leaks in a building envelope, open doors and windows and mechanical ventilation. To better understand total air pollution exposure, we utilized indoor and outdoor air quality data along with household characteristic survey data from an urban birth cohort study in Colorado [17] to evaluate three variable selection and/or penalization techniques to build a predictive model of indoor BC. Our study objective is to inform epidemiologic studies on environmental influences on early childhood health by investigating how indoor and outdoor BC are related and what additional factors are important in predicting indoor BC concentrations.

## 2. Methods

### 2.1. Study population

Healthy Start is a longitudinal pre-birth cohort study of ethnically diverse mother-infant dyads recruited at obstetrics clinics at the University of Colorado Hospital, which serves a nine-county region of Metropolitan Denver. Full descriptions of the study and recruitment strategy can be found in Harrod et al. [18]. Recruitment of pregnant women who had not yet reached 24 weeks of gestation, took place between 2009 and 2014, with the last birth occurring in September 2014. Participants attended follow-up visits through pregnancy, delivery and into early childhood of the offspring. Eligibility criteria included age > 16 years old, singleton pregnancy and no history of chronic disease such as diabetes, cancer, asthma, treatment with steroids, or medication-dependent psychiatric illness. Those with a history stillbirth or preterm birth (prior to 25 weeks of gestation) were also excluded. Participants reported their place of residence, age, race/ethnicity, education completed and the number of previous pregnancies at time of enrollment. A total of 1410 mother-child dyads were enrolled from whom data on pre-natal and perinatal outcomes were collected via questionnaire. Additional funding has allowed follow-up of the children and mothers over time to investigate the development of obesity and diabetes, in addition to other health endpoints including neurocognitive and respiratory outcomes. The study protocol was approved by the Colorado Multiple Institutional Review Board and all participants provided informed consent.

### 2.2. Study area and sampling locations

At the time of the study, the majority of Healthy Start participants lived primarily in Denver, Arapahoe and Adams Counties. As such, the community ambient air pollution monitoring campaigns were designed to estimate ambient air quality in these counties, primarily within the Interstate 470 loop that surrounds the Denver metropolitan area (sampling maps previously shown in Martenies et al. [8]) Monitoring sites were selected using a stratified sampling approach as described by Matte et al. [7]. to determine optimal monitor number and placement to represent regional TRAP sources and sinks. Briefly, the study region was overlaid with a 300 m x 300 m grid to stratify the area based on common sources of ambient PM2.5 (e.g., building density, road density, and traffic density) and to identify areas without coverage by central site monitors operated by the Colorado Department of Public Health and the Environment [8]. Residential monitoring locations were selected to optimize coverage of the study area and oversampled to reflect areas likely to be high sources of PM, while also considering the geographic location of the participants’ residences. We deployed 52 Ultrasonic Personal Air Samplers (UPAS; Access Sensor Technologies, Fort Collins, CO) across the study area that included Healthy Start participants’ residences in addition to public sites. We conducted spring (Campaign 1), summer (Campaign 2) and winter (Campaign 4) campaigns (May 8–July 3, 2018; July 10–August 27, 2018; January 22–March 12, 2019, respectively) to capture seasonal variability. Community ambient air quality sampling also took place in the fall (Campaign 3: October 10–November 11, 2018) but household indoor air was not sampled at that time. Once we identified areas within the predefined boundary for the model, we overlayed these locations with geocoded Healthy Start participant addresses to identify potential participants for this study. Families who had previously consented to be contacted about future research opportunities were invited to participate in this study. We stratified our recruitment efforts by region so that we could include participant homes across the study area. Once a potential participant expressed interest, we further screened their homes to ensure that we could suitably place our monitors both indoors and outdoors at their homes for the full sampling periods. Paired residential samples were measured during weeks 1 or 2 of each campaign.

### 2.3. Study design

Our main goal for this analysis was to gain an improved understanding of factors that are associated with BC levels inside homes. The objectives of this analysis were to investigate factors that are associated with BC levels inside homes, assess the seasonality of BC levels indoors and outdoors and build a predictive model for indoor and outdoor BC concentrations given the available set of housing and ambient environmental covariates. Within the scope of the prediction modeling, we sought to better understand which factors were predictive of indoor BC levels. We enrolled a panel of households to participate in seasonal repeated sampling of ambient and indoor air quality along with household characteristics to quantify these relationships. Residential enrollment was influenced by both the number of willing participants and the practical considerations of executing a large, multi-season, multi-week, field campaign [8]. Our number of households sampled is slightly greater than other studies that investigated BC infiltration [19, 20]. We were also interested in evaluating seasonal differences in BC concentrations, which are expected to change both indoor and outdoor due to fireplace/woodstove use, cooling methods, wildfires and seasonal weather pattern variations.

### 2.4. Participant home visits

**Fig. 1.**
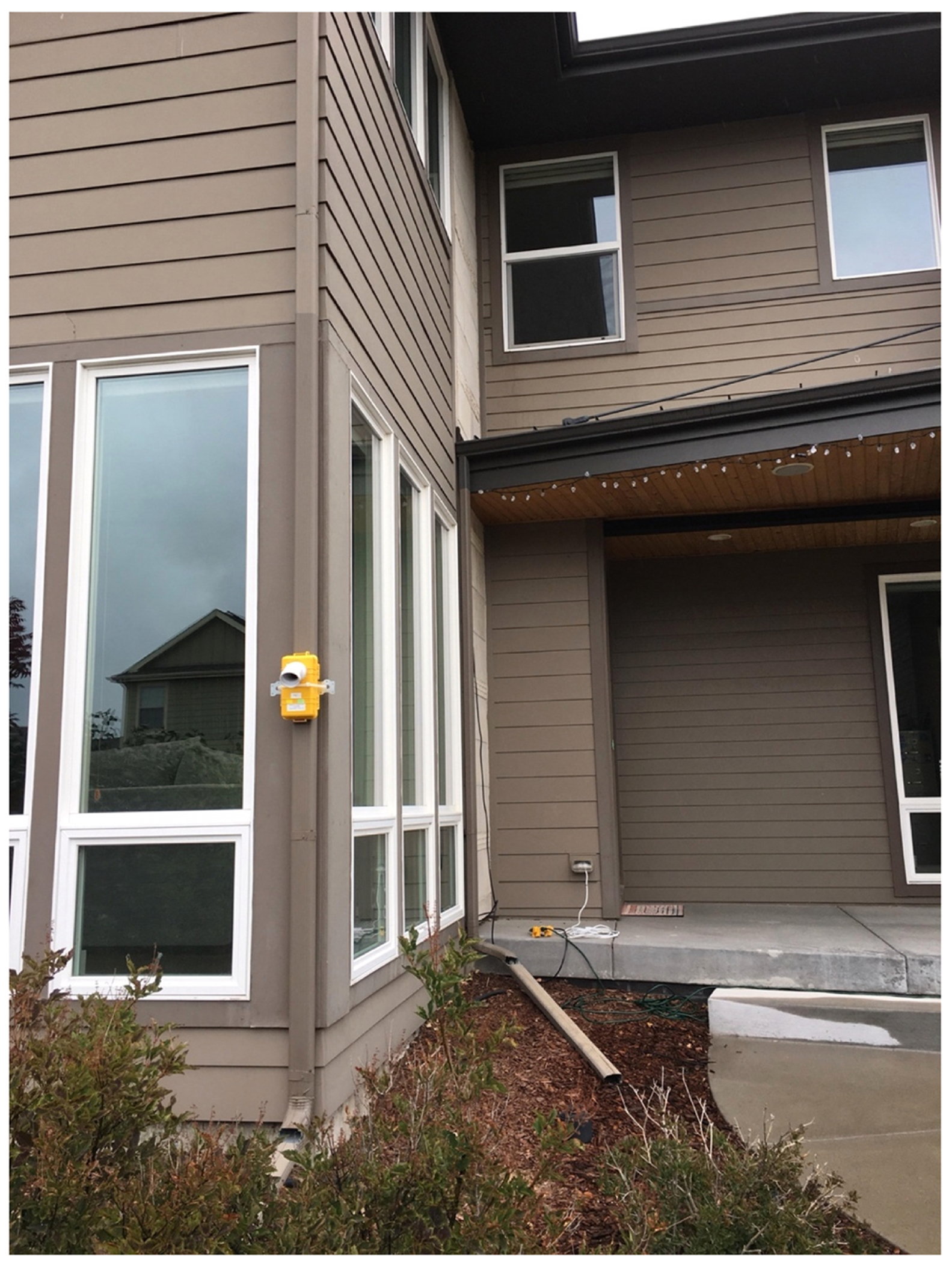
UPAS with weather resistant enclosure mounted to participant home.

A total of 27 households of Healthy Start participants enrolled in an ancillary study to host two UPAS for indoor and outdoor measurements (a study design summary is presented in Table 1). Field team members visited each home at a pre-scheduled date and time to install air quality samplers inside and outside of the participants’ homes. The filter-based UPAS allowed us to capture PM2.5 and constituents, including BC, metals and reactive oxidative species (ROS). Each outdoor UPAS was outfitted with a custom protective case and an external battery to extend run time to meet a 5-day target sampling period. Indoor monitors were set up in a shared space such as a living room or family room to capture typical indoor concentrations in spaces where occupants spent time. If the selected room had an outer wall, the outdoor monitor would be collocated on the other side of the wall. The field team avoided placing UPAS in rooms that had little foot traffic, were near inflow/outflow vents or air conditioning units or were near other PM2.5 sources such as cooking sources in the kitchen, or bedrooms. Outdoor UPAS were installed at adult breathing zone height or slightly above if risk of theft or tampering was perceived to be a concern (Fig. 1). Lamps, fence posts, porch beams and gutters were some of the items on which UPAS were attached via zip tie. Indoor samplers remained in the participants’ homes for 5 days, while outdoor UPAS stayed for the 7 week duration of the outdoor sampling campaign; filters were collected weekly at the end of each 5-day sampling period. Participants were compensated $25 for hosting a monitor and could receive a maximum of $75 in compensation provided for full participation in all three campaigns (spring, summer and winter).

### 2.5. Survey methods

During study visits, the adult study participant in the house responded to an English language questionnaire relating to household characteristics and occupation. The survey, adapted from the EPA’s BASE study [21] was designed to gather information about household characteristics that may influence indoor air quality, such as housing type, flooring, heating and cooling methods. Participants were also asked about their occupation and smoking status. Survey data were collected and managed using REDCap [22,23] electronic data capture tools hosted by the Colorado Clinical and Translational Sciences Institute.

### 2.6. Estimates of indoor BC concentration

#### 2.6.1. Quantification of PM and BC

The UPAS employs a size-selective cyclone inlet to filter out particulate matter of > 2.5 μm in diameter. For this analysis we used 5.5- day sampling period measurements from participants’ homes that hosted paired indoor and outdoor UPAS monitoring to collect BC and PM2.5 measurements during the spring, summer and/or winter campaigns. We collected particulate samples on polytetrafluoroethylene filters (MTL Corporation, Minneapolis, MN). UPAS flow rate was set to 1 L/min and units were run at an 80 % duty cycle to prolong battery life. Filters were pre-and post-weighed using the Automated Air Analysis Facility (AIRLIFT) at Colorado State University [24].

BC analysis methods for this project have been previously described in Martenies et al. [8]. Briefly, filters used to collect PM2.5 samples were analyzed for BC using SootScan Model OT21 transmissometer (Magee Scientific, Berkeley, CA); we used a previously established protocol described by Ahmed et al. [25] to calculate the mass of BC on each filter. Particle light absorption is calculated by measuring the amount of light attenuated when passed through a sampled filter; BC absorbs light strongly at the 880 nm wavelength. Attenuation at 880 nm was converted to BC density (μg cm^−2^) by dividing the attenuation by the mass absorbance cross section σ880 (m^2^ g^−1^). The final mass of BC on the filter was obtained by multiplying the BC density by the filter sampling area (α = 7.065 cm^2^). We used a value of 4.2 for the mass-absorbance cross section based on analysis by Presler-Jur et al. [26].

We deployed field blanks during each sampling campaign to assess potential contamination of filters. Measurements of BC from Campaign 1 and 2 showed a negative bias (mean BC measurements were – 4.0 μg and – 2.6 μg, respectively, with coefficients of variation (CV) < 25 %); therefore, BC measurements from these campaigns were blank corrected [8]. The limit of detection (LOD) value for BC was 1.41 μg based on the lower limit for the SootScan (0.2 μg/cm^2^) and the standard area of our filters (7.065 cm^2^). Filters below LOD, as well as filters with evidence of contamination (i.e., difference in pre- and post-sampling filter PM2.5 mass exceeded 1000 μg) were removed from the analysis.

Variability of BC measurements was evaluated at each site by campaign. As described in Martenies et al. [8], we expected to see higher variability in winter outdoor BC measurements due to the effect of cold on the sampling equipment (i.e., battery life sometimes decreased in the presence of lower ambient temperatures). The coefficient of variation was used as our criterion to evaluate variability in UPAS measurements collected at each site by campaign. Any observation that had values that exceeded the coefficient of variation for BC (0.30) during the sampling campaign was dropped.

### 2.7. Data analysis

To test for significant differences in BC concentrations across seasons, we used the Kruskal-Wallis rank sum test. We used Wilcoxon rank sum tests to evaluate the difference between indoor and outdoor BC concentrations across the entire study period, as well as differences between seasons. Wilcoxon signed rank tests were also applied to evaluate the difference between mean indoor and outdoor BC concentrations for the same season. Significance was determined using a 0.05 alphalevel test.

To understand the relationship between indoor and outdoor BC concentrations and build a predictive model, we investigated three regression modeling techniques and evaluated their performance using cross-validation. The first predictive modeling approach we used was to fit an evidence-based model with multiple linear regression, based on factors associated with indoor BC concentrations that have been previously described in the literature including infiltration of outdoor particulate matter, wood burning, seasonal change and residential building characteristics [19]. Factors examined in the modeling process are listed in Table 2 and include ambient and indoor air quality (PM2.5, BC) and housing characteristics (number of rooms, home ownership, presence of hard flooring, gas appliances, window AC use, presence of pets in home, dirty (BC producing) supplemental heat use, and smoking).

The other two modeling approaches were penalized regression models: LASSO (least absolute shrinkage and selection operator) and Ridge regression; both are machine learning techniques that were chosen for their interpretability and feature selection [27]. LASSO and Ridge, also known as L1 and L2 regularization, respectively, aim to mitigate over-fitting by introducing a penalty parameter that increases bias, thereby reducing variance. In this context, bias refers to the model’s inability to capture the true relationship between the variables, while variance represents the difference in fits across data sets [27]. The ideal algorithm will have low bias and low variance, producing consistent predictions across data sets.

Both Ridge and Lasso regression use a penalty parameter (λ_ridge_ and λ_lasso_) to penalize regression coefficients, but they differ in their penalty functions. In linear regression, the cost function is the average error of N-samples in the data, typically represented by the root mean squared error (RMSE) or mean squared error (MSE). The cost function for Ridge regression is the sum of squared residuals plus the penalty term:

(Eq. 1) $$\sum_{i=1}^{N}{\left(y_{i}-\beta_{0}-x_{i}^{\prime }\beta \right)}^{2}+\lambda_{ridge}\sum_{p=1}^{P}\beta_{p}^{2}$$

The amount of penalty is determined by λ_ridge_, which is selected through cross validation. If λ_ridge_ = 0, then the cost function is the same as the ordinary least squares (OLS) linear regression model; if λ_ridge_ > 0, it shrinks the regression coefficients towards zero [28].

However, Ridge regression never reduces a coefficient to zero, only to near zero; thus, it decreases the complexity of the model but does not reduce the number of variables. Therefore, Ridge performs better when most variables in the model are “useful” or provide information on the relationship of prediction variables and outcome [29]. Additionally, Ridge regression is beneficial for improving predictions with small samples sizes, as it makes predictions less sensitive to training data by increasing bias on the initial fit [30].

The cost function for Lasso regression is the sum of squared residuals plus the sum of the absolute value of the estimated regression coefficients:

(Eq. 2) $$\sum_{i=1}^{N}{\left(y_{i}-\beta_{0}-x_{i}^{\prime }\beta \right)}^{2}+\lambda_{lasso}\sum_{p=1}^{P}\left|\beta_{p}\right|$$

As with Ridge regression, the severity of the penalty for the Lasso is determined by the penalty parameter, λ_lasso_. However, as λ_lasso_ increases, the coefficient values will decrease until they reach zero. Thus, Lasso reduces the number of variables in the model, making it a viable option for variable selection. This approach may be helpful when working with datasets containing many noise variables or when there are too many predictor variables to have prior knowledge of which might be associated with the outcome. We hypothesize that the Ridge and LASSO will obtain improved predictive performance over the OLS model due to regularization.

For both models, we used two functions from the R package ‘glmnet‘, ‘lambda.min‘() and ‘lambda.ls‘(), to identify the penalty parameters [31]. The ‘lambda.min‘() function determines the penalty that obtains the minimum mean cross-validated error, while ‘lamda.ls‘() obtains the largest penalty parameter such that the error is within 1 standard deviation of the cross-validated errors for ‘lambda.min‘(). With these two approaches for selecting the penalty parameter, two models each were fit for the Ridge and LASSO.

For our analysis, we implemented leave-one-out cross-validation (LOOCV), which is useful for building predictive models with small datasets as it allows for the smallest amount of data removed from the training set in each iteration (i.e., only one observation). The final model was chosen based on the lowest mean square predictive error (MSPE). A multiple linear regression model was then fit for inference.

Prior to modeling, indoor BC concentrations were log transformed. All data cleaning and analyses were conducted in R Statistical Software (v.3.6.2 R Core Team, 2019) [32], primarily using the ‘tidyverse‘[33] and ‘glmnet‘[34] packages.

## 3. Results

### 3.1. Measurements and housing characteristics

A total of 27 distinct homes were sampled over the three campaigns. Additional households were recruited to replace two that were lost to follow-up during the spring or summer campaigns. We collected a total of 73 indoor/outdoor pairs (spring = 25, summer = 24, winter = 24) of residential filters.

Nine filter pairs were removed from analysis due to outdoor PM2.5 concentrations below LOD (n = 8) or potential contamination (PM mass > 1000 ug, n = 1). Additional pairs (n = 10) were removed that had indoor PM2.5 concentrations below LOD or a BC coefficient of variation > 0.30 (n = 8, all from winter campaign). Due to high variability and temperature related instrument performance issues in the winter samples, all winter data (n = 24) were excluded from analysis.

Following data cleaning, 39 measurements from 27 homes remained for the final analysis. Table 2 presents summary statistics of the homes used in the study along with median BC concentrations and Wilcoxon rank-sum p-values for each covariate. A univariable linear model was used to investigate the relationship between number of rooms in a home and median BC concentration (p = 0.67). We observed a significant association between housing characteristics and median BC concentration with presence of pets in the home (p = 0.03). Carpet was removed from further analysis due to the lack of variation (homogeneity) in the survey answers; dirty supplemental heat was removed due to homogeneity and the exclusion of paired winter data in the modeling process.

Table 3 presents sample measurement data stratified by season. The median indoor BC concentration was 0.99 μg/m^3^ (0.25, 2.25) during the spring, 1.00 μg/m^3^ (0.05, 4.65) during the summer, and 0.37 μg/m^3^ (0.09, 0.87) during the winter with an overall median of 0.90 μg/m^3^. Homes monitored during the spring had a ratio of median indoor to median outdoor BC concentrations equal to 0.99, while homes monitored during summer had a ratio of median indoor to median outdoor BC concentrations equal to 0.76. Outdoor winter BC data were excluded from the analysis due to poor quality.

Using a Kruskal-Wallis rank-sum test, median indoor BC concentrations demonstrated a significant difference (p = <0.01) between Spring, Summer and Winter seasons (Table 4). There was no significant difference in outdoor (p = 0.86) BC concentrations between the Spring and Summer seasons, with winter data excluded due to quality issues. We also found no significant difference between paired indoor and outdoor BC concentrations by Spring and Summer seasons using a Wilcoxon signed-rank test (Spring; p = 0.56, Summer; p = 0.18; Table 5).

### 3.2. Model performance

**Fig. 2.**
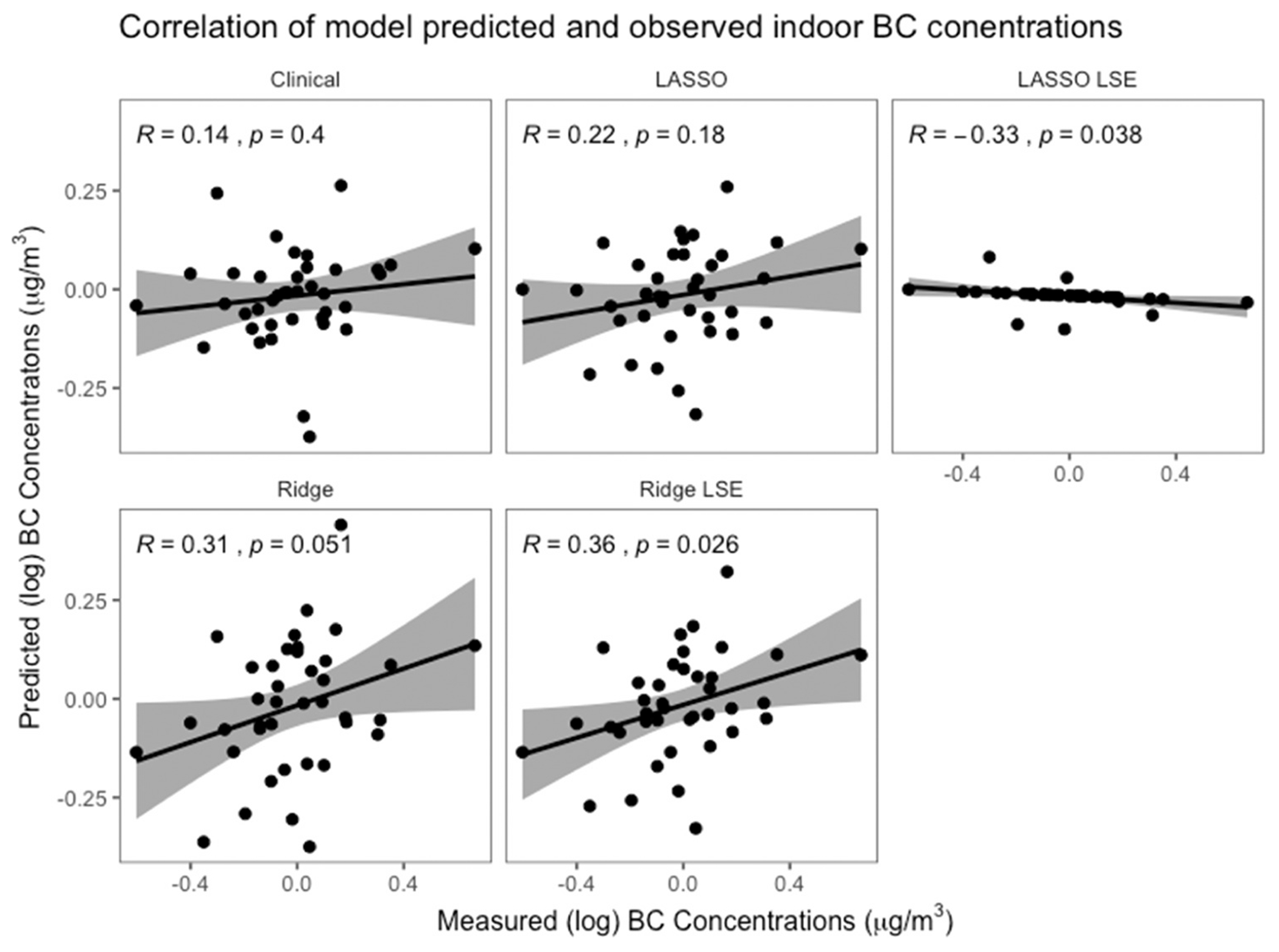
One-to-one correlation plots of Clinical, LASSO, LASSO LSE, Ridge Regression and Ridge Regression LSE predictions of log indoor BC concentrations for all measurements sites, spring and summer campaigns.

In terms of predictive performance, the evidence-based model had an MPSE of 0.054 and estimates for the four covariates selected to predict indoor BC a priori are as follows: outdoor PM2.5 (β = 0.027), outdoor BC (β=0.052), gas appliance in home (β = 0.051) and season (β = 0.027). The LASSO model obtained an MPSE of 0.064 and selected three predictors: outdoor PM2.5, presence of pets in home, and presence of greater than two types of hard flooring. The Ridge least-squared errors (LSE) model obtained the best predictive performance with a MPSE of 0.050 (Table 6). Since Ridge does not eliminate covariates and cannot be used specifically for feature selection, we chose the three covariates selected by LASSO for our final inference model: outdoor PM_2.5_ concentration, presence of pets in home, and presence of greater than two types of hard flooring. A linear regression model with these three covariates was fit for inference (Table 7). The R^2^ for the final model was 0.28. Households that had more than two types of hard flooring had a 0.09 (95 % CI: – 0.49, 0.22) unit increase in log-transformed indoor BC concentrations compared to homes that had one or fewer types of hard flooring. Households with pets in the home had a 0.12 (95 % CI: – 0.01, 0.26) unit increase in log-transformed indoor BC compared to homes with no pets. Fig. 2 shows correlation plots regressing model predicted log BC concentrations on observed log BC values.

To visualize what percent of the variation was not explained by outdoor BC alone, we calculated the coefficient of partial determination [35], or partial R^2^, for the remaining variables (Table 8). Starting with outdoor BC concentration as the only predictor variable produced an R^2^ of 0.09. The final model which included outdoor BC, outdoor PM_2.5_, hard flooring and pets in home had an R^2^ of 0.27.

## 4. Discussion

Using three predictive modeling approaches, including Ridge and LASSO regression, we developed a model from our data to predict indoor BC concentrations using housing characteristics and outdoor BC and PM2.5 concentrations. Our final model explained 28 % of the variability in measured indoor BC. The three predictors in the final model were outdoor PM2.5, presence of more than two types of hard flooring and presence of any pets in the home. We hypothesized that the presence of more than two types of hard flooring may indicate fewer carpeted surfaces. Carpeted surfaces might capture more BC, which is then filtered out during vacuuming. Additionally, hard flooring could enhance the resuspension of BC compared to carpet. Pets in the home may predict higher indoor BC concentrations due to increased ventilation, as pet owners might open doors more frequently to let their pets outside. Furthermore, the fur of pets could transport particles from outdoors into the home.

Our results are comparable to a previous study by Baxter et al. that used publicly available central site monitor and GIS data, property assessment records and questionnaire responses from lower socioeconomic status (SES) households to predict indoor elemental carbon (EC) in a subset of participants of a prospective birth cohort in Boston, MA. Sampling was conducted in two seasonal periods – heating (December–March) and non-heating (May–October); their linear modeling strategy resulted in a model R^2^ = 0.32 [36]. Although indoor sources were identified for PM2.5 (cooking time) and NO_2_ (stove usage), no indoor source was identified for EC. The truck traffic indicator proved to be an important factor in the modeling approach but was strongly modified by the variable “windows open” which was collected from the survey question asking if participants did or did not open their windows. The R^2^ of our model was much lower than that in a study by Isiugo et al., where researchers developed a predictive model for BC that explained 78 % of the variability in indoor BC concentrations. The 23 sampling sites included houses and apartments belonging to a cohort of subjects from another ongoing study focused on the efficiency of air cleaners in removing indoor particles [37]. In the Isiugo et al. study, presence or absence of electrostatic/HEPA HVAC filters was one of the most important factors influencing indoor BC concentration – homes with filters had an indoor/outdoor BC ratio of 0.18 while homes without had a ratio of 0.62. Both studies point to the importance of correctly capturing the heterogeneity in outdoor BC concentrations as BC tends to be dominated by outdoor sources.

The three different predictive modeling strategies were quite similar with respect to MPSEs ranging from 0.050 with Ridge LSE to 0.064 with LASSO. Ridge is a useful modeling tool when working with multiple covariates that offer some explanation regarding the variability of the outcome while LASSO is better at eliminating noise variables [38]. In this study, many of the covariates can at least partially explain the variability of the outcome of indoor BC concentrations. Ridge is the preferred method to address multi-collinearity, or correlation between predictor variables. LASSO will generally remove some of the collinear variables in the model, and Ridge will keep all collinear variables while shrinking some of their coefficients. Although Ridge does not eliminate predictor variables and reduce model complexity, it may be the better statistical tool for predicting indoor BC concentrations due to the information on covariates that is retained.

### 4.1. Applications of a predictive model

A predictive model for indoor BC would be a helpful tool to support public health risk assessment objectives and to reduce exposure misclassification in studies that assign individual BC exposure levels. Cumulative exposure is a combination of indoor concentration, outdoor concentration and time-activity patterns related to those environments. Collecting indoor BC measurements is a time and cost burden that could potentially be avoided by collecting household survey data and information on time-activity patterns. However, results from this study, as well of those from Baxter et al. and Isiugo et al., demonstrate that building characteristic data and other information such as distance to a highway may not explain enough variability in indoor BC measurements. In addition, estimating only residential exposure does not capture exposure from other microenvironments including school, work, and commuting. With the increasing availability of reliable, low-cost monitors, personal monitoring may be a more robust and feasible method to estimate individual exposure to BC. Additional questions regarding indoor BC sources could be addressed by placing a sampler inside the home during the personal monitoring period. Improvements in monitor technology, such as GPS functionality and real-time PM sensors will help us better understand exposure by microenvironment.

### 4.2. Limitations

We had limited comparable data on seasonality as winter outdoor data were excluded from the analysis. Shortened runtimes and other performance issues suggest that the poor data quality of winter outdoor BC measurements may be explained in part by impaired function and reliability and insufficient weatherproofing of our monitors in cold weather. Thus, the only two seasons we had for modeling were summer and spring (“non-heating” seasons), which overall did not show a significant difference in indoor or outdoor BC concentrations. Other studies have shown outdoor BC to be negatively correlated with temperature and wind speed with higher average concentrations found during the winter [8,39].

Information on housing characteristics was limited and did not include variables that were shown to be informative in an indoor BC predictive modeling study by Isiugo et al. such as kitchen stove ventilation status, candle use during the study period and type of HVAC filter used in the home. For future studies, we would include more specific information on type of ventilation used (open windows, fans, HVAC, etc.) as well as percent of flooring area covered in either carpet or hard floors such as tile or concrete. Although rate of infiltration and exfiltration through the building envelope are important variables when considering predictors of indoor air quality, gathering that information directly would not be feasible for large-scale studies since it would require individual assessment at each participant residence. However, estimates of infiltration can be discerned from existing models including factors such as building age, size and climate zone [40]. Distance of homes from nearby roads with high vehicular, particularly diesel traffic, may also modify indoor BC concentrations and will be incorporated into future modeling strategies [41,42].

Although we recognized the importance of household behaviors, such as cooking and use of incense and or candles, on indoor air quality, our survey and study design were not adequate to capture these activities during the monitoring campaign [43]. Part of this limitation was due to our use of integrated filter-based measurements over a long period of time (5.5 days) where short-term behavioral changes could not be linked to averaged BC levels. Further, the Colorado Front Range, including the Denver metro area, is known to experience high ozone concentrations due to unique topography and atmospheric chemistry concerns. Colorado’s numerous sunny days along with an abundance of ozone source chemicals such as NOx and VOCs lead to many days where Denver ozone levels violate EPA ambient air quality standards [44]. In addition, unique airflow patterns can trap pollution near ground level. Results from an analysis of the Healthy Start cohort data in 2020 show limited evidence of associations with prenatal TRAP exposure and infant body composition, yet did show that third trimester ozone exposure was associated with greater adiposity at early postnatal follow-up visits [45]. Taken together, air pollution factors unique to our study area in addition to challenges related to lack of real time data may have affected our ability to build models with higher predictive power. We have updated this strategy and are now conducting sampling using a UPAS with real time measurements along with a household survey that captures behavior and indoor sources of PM [46].

The Ridge LSE model had the strongest predictive performance, and we would suggest using that model if the main objective is to predict indoor BC with similar factors as we used in our study. We could further develop our model by investigating interaction terms or potential nonlinear relations.

## 5. Summary and conclusions

The current analysis identified predictors of indoor air concentrations for black carbon using paired ambient BC and PM2.5 concentrations and questionnaire data relating to household characteristics. The use of LASSO and Ridge penalization models helped us fit the strongest predictive model given the available covariates as well as narrow down selection of variables for an inference model.

Our study provides some direction regarding how survey data and ambient PM2.5 and BC concentrations can be utilized in population studies to predict residential indoor exposures in the absence of individual household measurements. We have demonstrated that information on household characteristics obtained by survey can enhance ambient monitoring data in determining predictors of indoor BC exposure.

## Figures and Tables

**Table 1:** Study design summary.

**Households**	27 Healthy Start participants in Denver Metro area
**Campaign Dates**	(1) 5/18/18 – 7/3/18, (2) 7/10/18 – 8/27/18, (3) 1/22/19–3/1/19
**Sampler Deployment**	Two UPAS per household (indoor and outdoor)
**Measurements**	PM2.5, BC, metals, ROS
**Indoor UPAS Placement**	Shared spaces (living room, family room), avoiding low foot traffic areas, vents, kitchens
**Outdoor UPAS Placement**	Adult breathing zone height, secured to lamps, fence posts, porch beams, gutters
**Sampling Duration**	Indoor: 5 days; Outdoor: 7 weeks
**Filter Collection**	Weekly, at the end of each 5-day sampling period
**Participant Compensation**	$25 per monitor, up to $75 for full participation in all three campaigns
**Survey**	Questions relating to household characteristics that influence indoor air quality

**Table 2:** BC concentration by household characteristic. Using spring and summer data, P-values were calculated using Wilcoxon rank-sum test, except for number of rooms, where a linear model with log-transformed BC values was used.

	Overall (n = 39)	Median BC concentration (μg/m_3_)	p-value
**Number of rooms in home**			
Mean (SD)	9.82 (3.36)	n/a	0.67
**Single family home**			
no	8 (20.5 %)	0.65	
yes	31 (79.5 %)	1.00	0.12
**Participant owns home**			
no	12 (30.8 %)	1.05	
yes	27 (69.2 %)	0.92	0.54
**Carpet in any rooms**			
no	1 (2.6 %)	2.05	
yes	38 (97.4 %)	0.97	0.15
**Hard flooring (> two types)**			
no	18 (46.2 %)	0.87	
yes	21 (53.8 %)	1.09	0.06
**Gas appliances in home**			
no	22 (56.4 %)	0.99	
yes	17 (43.6 %)	0.96	0.57
**Window AC in home**			
no	35 (89.7 %)	0.92	
yes	4 (10.3 %)	1.05	0.37
**Smoker in home**			
no	34 (87.2 %)	0.91	
yes	5 (12.8 %)	1.09	0.26
**Pets in home**			
no	19 (48.7 %)	0.83	
yes	20 (51.3 %)	1.07	0.03
**Dirty supplemental heat ever used (wood stove, wood fireplace, kerosene gas)**			
no	35 (89.7 %)	1.00	
yes	4 (10.3 %)	0.76	0.20

**Table 3:** Median BC and PM_2.5_ measurements (μg/m^3^) by season.

	Spring (n = 21)	Summer (n = 18)	Winter (n = 21)	Overall (n = 60)
**Indoor BC**				
**Median [Min, Max]**	0.99 [0.25, 2.25]	1.00 [0.05, 4.65]	0.37 [0.09, 0.87]	0.90 [0.09, 4.65]
**Outdoor BC**				
Median [Min, Max]	1.01 [0.36, 2.12]	1.15 [0.052, 2.57]	*	1.10 [0.052, 2.57]
**Indoor PM_2.5_**				
Median [Min, Max]	13.8 [6.13, 32.4]	11.9 [2.83, 75.1]	7.06 [2.23, 22.39]	9.87 [0.17, 75.06]
**Outdoor PM_2.5_**				
Median [Min, Max]	9.15 [7.19, 17.1]	10.3 [3.48, 18.6]	*	9.62 [3.48, 18.6]
**Indoor/outdoor BC ratio**	0.990	0.757	*	0.843

* Outdoor winter data excluded due to poor quality.

**Table 4:** Kruskal-Wallis rank-sum test comparing median seasonal concentrations of BC.

			P-value
Summer Indoor BC	Spring Indoor BC	Winter Indoor BC	< 0.01
Summer Outdoor BC	Spring Outdoor BC	*	0.88

* Outdoor winter BC data excluded due to poor quality.

**Table 5:** Wilcoxon signed-rank test comparing paired BC samples by season.

		P-value
Spring Indoor BC (median = 1.01)	Spring Outdoor BC (median = 1.00)	0.56
Summer Indoor BC(median = 0.87)	Summer Outdoor BC (median = 1.15)	0.18

**Table 6:** Model summary – comparison of coefficients from LASSO, ridge and evidence-based models. For ridge and LASSO, minimum of mean cross-validated errors is used to calculate λ, while LSE models use the largest value of λ such that the error is within 1 standard error of the cross-validated minimum.

Covariates/Predictors					
	LASSO	LASSO LSE	Ridge	Ridge LSE	Evidence-based
**Outdoor PM_2.5_**	0.013	2.15 × 10^−17^	0.016	0.014	0.027
**Indoor PM_2.5_**			0.004	0.003	
**Outdoor BC**			0.045	0.041	0.050
**Number of rooms in home**			− 0.003	− 0.002	
**Single family home**			0.084	0.066	
**Participant owns home**			− 0.054	− 0.046	
**Hard flooring (> 2 types)**	0.033		0.127	0.092	
**Gas appliance in home**			− 0.058	− 0.022	0.051
**Window AC in home**			0.078	0.052	
**Smoker in home**			− 0.044	− 0.013	
**Pets in home**	0.064		0.119	0.094	
**Season - spring**			− 0.006	− 0.009	
**Season - summer**			0.006	0.009	0.046
**MPSE**	**0.064**	**0.053**	**0.056**	**0.050**	**0.054**

* MPSE = mean squared prediction error, LSE = least squared error.

**Table 7:** Summary of linear regression model with covariates selected by LASSO, R^2^ = 0.28.

	Regression estimate (ß)	Standard error	P-value	95 % Confidence Interval
**Intercept**	− 0.313	0.356	0.385	(− 1.035,0.409)
**Outdoor PM_2.5_**	0.117	0.035	0.002	(0.045,0.188)
**Hard flooring (> than two types)**	0.222	0.211	0.300	(− 0.207,0.652)
**Pets in home**	0.267	0.208	0.208	(− 0.155,0.689)

**Table 8:** Coefficients of partial determination (partial R^2^) for BC model including outdoor BC.

	R^2^	P-value
**Outdoor BC**	0.09	0.03
**Outdoor BC + Outdoor PM_2.5_**	0.20	0.01
**Outdoor BC + Outdoor PM_2.5_ Hard flooring**	0.23	0.01
**Outdoor BC + Outdoor PM_2.5_ + Hard flooring + Pets in home**	0.27	0.01
